# The effect of maternal undernutrition on the rat placental transcriptome: protein restriction up-regulates cholesterol transport

**DOI:** 10.1186/s12263-016-0541-3

**Published:** 2016-10-12

**Authors:** Zoe Daniel, Angelina Swali, Richard Emes, Simon C Langley-Evans

**Affiliations:** 1 School of Biosciences, University of Nottingham, Sutton Bonington, Loughborough, LE12 5RD UK; 2 School of Veterinary Medicine and Science, University of Nottingham, Sutton Bonington, Loughborough, UK; 3 Advanced Data Analysis Centre, University of Nottingham, Sutton Bonington, Loughborough, UK

**Keywords:** Placenta, Transcriptome, Pregnancy, Protein restriction, Undernutrition, Cholesterol

## Abstract

**Background:**

Fetal exposure to a maternal low protein diet during rat pregnancy is associated with hypertension, renal dysfunction and metabolic disturbance in adult life. These effects are present when dietary manipulations target only the first half of pregnancy. It was hypothesised that early gestation protein restriction would impact upon placental gene expression and that this may give clues to the mechanism which links maternal diet to later consequences.

**Methods:**

Pregnant rats were fed control or a low protein diet from conception to day 13 gestation. Placentas were collected and RNA sequencing performed using the Illumina platform.

**Results:**

Protein restriction down-regulated 67 genes and up-regulated 24 genes in the placenta. Ingenuity pathway analysis showed significant enrichment in pathways related to cholesterol and lipoprotein transport and metabolism, including atherosclerosis signalling, clathrin-mediated endocytosis, LXR/RXR and FXR/RXR activation. Genes at the centre of these processes included the apolipoproteins ApoB, ApoA2 and ApoC2, microsomal triglyceride transfer protein (Mttp), the clathrin-endocytosis receptor cubilin, the transcription factor retinol binding protein 4 (Rbp4) and transerythrin (Ttr; a retinol and thyroid hormone transporter). Real-time PCR measurements largely confirmed the findings of RNASeq and indicated that the impact of protein restriction was often striking (cubilin up-regulated 32-fold, apoC2 up-regulated 17.6-fold). The findings show that gene expression in specific pathways is modulated by maternal protein restriction in the day-13 rat placenta.

**Conclusions:**

Changes in cholesterol transport may contribute to altered tissue development in the fetus and hence programme risk of disease in later life.

**Electronic supplementary material:**

The online version of this article (doi:10.1186/s12263-016-0541-3) contains supplementary material, which is available to authorized users.

## Background

The causes of chronic diseases of adulthood are complex. In addition to influences of adult lifestyle, such as dietary pattern, physical activity and the consumption of alcohol and smoking, the environment experienced during infancy and fetal life plays a critical role in establishing adult metabolic and cardiovascular phenotypes [1]. Early life exposure to poor nutrition (both under- and over-nutrition) can programme aspects of adult anatomy, physiology and metabolism [2, 3]. Risk of cardiovascular and metabolic disorders that emerge later in life may therefore already in place even before birth. Epidemiological studies which show relationships between proxy markers of poor nutrition in pregnancy and diseases including cardiovascular disease, type-2 diabetes and chronic kidney disease are supported by observations in animals [2, 4–6]. Manipulating either overall food supply or dietary composition such that one or more nutrients is limiting during pregnancy leads to permanent changes in organ structure and establishes a predisposition to ageing-related insulin resistance, cardiovascular dysfunction and renal disease [1].

We previously showed that exposure of the developing rat fetus to maternal undernutrition (both protein restriction and iron deficiency) up to day 13 gestation (full-term is 22 days) induced changes in renal morphology that may underpin the development of hypertension in later life [7]. These effects were associated with a number of changes in the expression of genes and proteins in the day 13 embryo, which were clustered around regulation of the cell cycle, the cytoskeleton and formation of clathrin vesicles [7, 8]. Whilst these processes within the embryo can be envisaged as contributing to remodelling of tissues and therefore permanent changes in the physiology of the animal, leading to later disease [9], they do not give an indication of what initiates these changes in response to maternal diet.

The placenta has long been recognised as having an important role in nutritional programming of later disease [10] either through dietary modulation of placentally derived hormones, dietary modulation of the placental transport of hormones [11] or variation in the delivery of key substrates to the developing fetus [12]. As such, it may be at the centre of the response to maternal undernutrition and the transfer of signals of adverse conditions from mother to fetus. Placental functions will vary with stage of development and the demands of the fetus. In this study, we have focused on the day-13 rat placenta. At this point, full development of the organ has not been completed, but all five basic placental layers are in place (myometrium, deciduum, giant trophoblasts, trophospongium and labyrinth; [13]. The tissue is rich in blood cells and glycogen cells but has not yet developed invasive vessels [13]. In the rat, maximum placental weight is not reached until day 16. We hypothesised that the established but immature placenta would show differential patterns of gene expression in response to maternal protein restriction. These patterns may give important clues as to how maternal nutrition at this stage of development may have long-term consequences for the fetus.

## Methods

This paper reports data from analysis of placentas collected in our previously published study of gene and protein expression in day-13 rat embryos [7]. Female virgin Wistar rats (Harlan, UK) were subjected to a 12 h light (08:00–20:00)-dark (20:00–08:00) cycle at a temperature of 20–22 °C with ad libitum access to food and water. At a weight of approximately 180–200 g, females were mated with stud males. After conception, determined by the presence of a semen plug on the cage floor, females were single-housed and animals were fed either a control 18 % (*w*/*w*) casein protein diet (control protein (CP)) or a 9 % (*w*/*w*) casein (low protein (LP)) diet until day 13 gestation (*n* = 8 per group). The LP diet was isocaloric relative to the control (see Additional file 1: Table S1 for composition of diets). To achieve a 50 % reduction in protein content of the LP diet, an additional 9 % carbohydrate was added. We have previously discussed the relative contributions of protein, carbohydrate and lipids to programming effects of the diet in detail [14–16]. During pregnancy, the animals were weighed and food intake was recorded daily. All animal work was performed under licence from the Home Office (UK) and complied with the Animals (Scientific Procedures) Act (1986). The project was approved by the University of Nottingham, Animal Ethics Committee.

On day 13 of gestation the rats were culled by CO_2_ asphyxia and cervical dislocation. Individual embryos and placentas were harvested. Tails were removed from embryos to establish sex. Tissues were snap frozen in liquid nitrogen and stored at −80 °C. PCR was used to verify presence or absence of the sex determining region-Y (SRY) gene in lysed embryo tail tissue [7]. This study used placenta only from male embryos and to generate the RNA samples for RNASeq analysis three placentas from the same litter were pooled. Only male embryos were selected to remove complications of sex from the analysis. Previous work has shown that the impact of maternal undernutrition upon long-term health of offspring is greater in males than in females [17–19]. Overall six samples per group were used for the analysis, with each sample representing three placentas associated with male embryos from a separate litter (18 placentas, 6 litters per group).

High-quality RNA was prepared from frozen tissue using Roche High Pure Tissue Kit according to the manufacturer’s instructions. Samples of high-quality RNA (RIN >6.0) were sent to Oxford Gene Technology (Begbrooke, Oxfordshire, UK) for polyA-enriched RNA sequencing using the Illumina TruSeq RNA sample prep kit v2 (Illumina, Little Chesterford, Essex, UK). With this kit, total RNA was captured using olido-dT coated magnetic beads and messenger RNA (mRNA) was fragmented and randomly primed. First strand complementary DNA (cDNA) was initiated from random primers, followed by second strand synthesis. After end repair, phosphorylation and A-tailing, adapter ligation and PCR amplification was performed to prepare the library for sequencing.

Sequencing was performed on the Illumina HiSeq2000 platform using TruSeq v3 chemistry. Read files (Fastq) were generated from the sequencing platform via the manufacturer’s proprietary software, and read level QC metrics were generated by FastQC http://www.bioinformatics.babraham.ac.uk/projects/fastqc/). Reads were processed through the Tuxedo suite [20] and mapped to their location using Bowtie version 2.o2 (http://bowtie-bio.sourceforge.net/index.shtml). Cufflinks v2.1.1 (http://cole-trapnell-lab.github.io/cufflinks/) was used to perform transcript assembly, abundance estimation and differential expression for the samples. RNASeq alignment metrics were generated using Picard tools (http://broadinstitute.github.io/picard/).

RNASeq was carried out on 12 samples with an average of 12279507 paired end reads per sample. A total of 11.63 gigabases of sequence data were read and aligned at high quality. The number of mapped reads per sample ranged from 3081828 to 17579532, and the proportion of mapped reads exceeded 99 % across all samples. The percentage of high-quality aligned bases was in excess of 98.5 and >96.5 % of reads were aligned in pairs.

Data was analysed using Cufflinks v2.1.1. A one-sided *t* test was used to determine the significant changes in gene expression (*P* value), and a Benjamini-Hochberg correction for multiple testing was also used (*q* value) as reported by Trapnell et al. [21]. Selection of genes identified as differentially expressed in the protein restricted group was based upon false discovery rate adjusted *q* values <0.05 (unadjusted *P* < 0.0005). Pathways and networks of interacting proteins enriched for differentially expressed genes were identified using ingenuity pathway analysis. Statistical enrichment is calculated by a right tailed Fisher’s exact test (IPA, QIAGEN Redwood City www.qiagen.com/ingenuity).

To further explore the differential expression data, we performed quantitative real-time PCR for 13 genes that were differentially expressed according to the RNASeq analysis. These included seven genes in the main pathways showing enrichment in the ingenuity analysis (ApoA2, ApoC2, Ttr, Fgg, Actg2, serpin G1 and Rbp4); Cubn and Mttp, which have functions closely related to those enriched pathways; and four genes that were shown to be differentially expressed in the protein restricted condition (Vil1, Gpc3, Muc13, Prf1). The PCR measurements were performed on the same RNA samples that were originally analysed through RNASeq. Total RNA (500 ng) was reverse transcribed using a cDNA synthesis kit (RevertAid RT Reverse Transcription Kit, Thermo Fisher) with random primers. Real-time PCR primers were designed using Primer Express software (version 1.5; Applied Biosystems) from the RNA sequence, checked using BLAST (National Center for Biotechnology Information) and were purchased from Sigma (UK). The primer sequences for these analyses are presented in Additional file 2: Table S2. Real-time PCR was performed on a Lightcycler 480 (Roche, Burgess Hill, UK) using the 384 well format. Each reaction contained 5 μl of cDNA with the following reagents: 7.5 μl SYBR green master mix (Roche), 0.45 μl forward and reverse primers (final concentration 0.3 μM each) and 1.6 μl RNase-free H_2_O. Samples were pre-incubated at 95 C for 5 min followed by 45 PCR amplification cycles (de-naturation, 95 C for 10 s; annealing, 60 C for 15 s; elongation, 72 C for 15 s). Transcript abundance was determined using a standard curve generated from serial dilutions of a pool of cDNA made from all samples. Expression was normalised against the expression of cyclophilin, which was shown to be unaffected by maternal diet in the RNASeq analysis and subsequently by PCR. The primer sequences for these analyses are presented in Additional file 2: Table S2. Data from real-time PCR measurements was tested using independent samples *t* tests. Ten of the targets were shown to be differentially expressed in the protein restricted group, confirming the RNASeq analysis.

## Results

The RNASeq analysis revealed differential expression of 91 genes in the day 13 rat placenta in response to maternal protein restriction. Of these, 24 were up-regulated and 67 were down-regulated. The full list of differentially expressed genes is provided in Table 1, and the full transcriptome analysis is available in Additional file 3: Table S3.

**Table 1:** Differentially expressed genes in rat placenta at d13 gestation

Gene ID	Locus	Expression (control)	Expression (LP)	Fold−change (log2)	*Q* value
Actg2	4:117732482-117747006	71.49	2.48	−4.85	0.00922
Gzmf	15:34696237-34707618	103.92	4.98	−4.38	0.00922
Gzmb	15:35195449-35198468	384.48	20.68	−4.22	0.00922
Nkg7	1:93813122-93814188	146.90	8.65	−4.09	0.00922
Prf1	20:28658366-28663866	171.89	10.39	−4.05	0.00922
Ccl5	10:71605790-71610330	59.82	4.15	−3.85	0.00922
Zbtb32	1:85635283-85637589	7.78	0.57	−3.77	0.04862
Asb2	6:127609501-127645492	5.26	0.46	−3.53	0.00922
Lama2	1:18203478-18885460	4.48	0.40	−3.49	0.00922
Cd96	11:56183624-56258356	11.16	1.03	−3.43	0.01702
MYH11_RAT	10:666714-776052	8.22	0.77	−3.42	0.00922
Rgs1	13:58121190-58125514	12.73	1.29	−3.31	0.00922
Ptprcap	1:206738734-206740893	13.28	1.57	−3.08	0.04148
LOC305103	13:88606173-88611105	120.74	14.38	−3.07	0.00922
D4ADB8_RAT	8:21210583-21221026	9.83	1.18	−3.06	0.00922
Cdh17	5:26047159-26099164	3.75	0.46	−3.02	0.02304
E9PSV0_RAT	20:4300723-4315876	10.67	1.34	−2.99	0.00922
Igfbp6	7:140885375-140890043	228.66	28.85	−2.99	0.00922
Col6a6	8:110793848-110892578	5.48	0.69	−2.98	0.00922
ADH1_RAT	2:235799456-235811584	69.42	9.27	−2.90	0.00922
Mcpt9	15:34541881-34544835	26.43	3.53	−2.90	0.00922
Lck	5:148707506-148718296	15.93	2.21	−2.85	0.00922
C1s	4:160736132-160748150	22.95	3.25	−2.82	0.00922
Pla1a	11:64099836-64137355	12.63	1.79	−2.82	0.00922
Sep1	1:186474714-186478580	11.10	1.58	−2.82	0.00922
COBA1_RAT	2:209996818-210193378	12.61	1.92	−2.71	0.02304
C1r	4:160712581-160729361	29.45	4.56	−2.69	0.00922
Q3MIE5_RAT	10:19207498-19660353	2.64	0.41	−2.67	0.00922
CLM8_RAT	10:104775859-104788927	15.09	2.50	−2.59	0.01702
Phf11	15:38444406-38477945	27.05	4.56	−2.57	0.00922
Sfrp4	17:53121424-53131513	42.94	7.23	−2.57	0.00922
Cytip	3:39893892-39921114	6.66	1.17	−2.50	0.02304
Rac2	7:116520065-116532482	38.51	6.90	−2.48	0.00922
C1qb	5:155647525-155653074	13.93	2.52	−2.47	0.02873
Coro1a	1:185852741-185857715	40.30	7.80	−2.37	0.00922
Aldh1a2	8:75692098-75771159	6.41	1.26	−2.35	0.00922
Pla2g2a	5:157654785-157657360	19.90	3.90	−2.35	0.02304
Smoc2	1:53165791-53295122	3.31	0.68	−2.29	0.04862
Rab27a	8:77798829-77861089	8.05	1.73	−2.22	0.00922
Serping1	3:67968807-67978102	39.91	8.61	−2.21	0.00922
D3ZXA0_RAT	15:38372728-38391822	21.10	4.62	−2.19	0.00922
RGD1565772	1:67630583-67648373	4.21	0.94	−2.17	0.00922
Rgs2	13:57890948-57894465	30.51	6.87	−2.15	0.00922
Psmb8	20:4786263-4789173	43.92	10.48	−2.07	0.00922
Tagln	8:48902208-48907693	99.68	23.75	−2.07	0.00922
C1qc	5:155656104-155659430	13.41	3.31	−2.02	0.04502
Prelp	13:46801474-46943977	12.67	3.13	−2.02	0.00922
LOC100365668	10:38109857-38110205	120.96	30.50	−1.99	0.00922
Fst	2:46542245-46550678	60.50	15.49	−1.97	0.00922
Ptprc	13:51246163-51357995	11.13	2.93	−1.93	0.00922
Selplg	12:43842267-43843560	11.84	3.10	−1.93	0.04862
Angpt4	3:142249114-142282307	14.42	4.07	−1.82	0.04502
Itgal	1:186561794-186598905	4.37	1.26	−1.80	0.02873
Fcer1g	13:87119465-87123902	78.98	23.70	−1.74	0.00922
Plek	14:97841598-97875052	18.70	5.63	−1.73	0.00922
Ccdc88b	1:209520223-209536201	3.50	1.06	−1.72	0.04502
Ifitm3	1:201198666-201199807	153.46	48.97	−1.65	0.01702
Pcolce	12:19672504-19678821	53.84	18.12	−1.57	0.04862
Plcg2	19:47875571-47947573	5.87	2.12	−1.47	0.03500
Prl8a7	17:44148436-44154238	43.87	15.87	−1.47	0.00922
Bgn	X:159380548-159391521	29.57	11.81	−1.32	0.02873
Cgm4	1:77441012-77453814	15.02	6.04	−1.31	0.02304
Pmp22	10:49305834-49335864	30.24	12.39	−1.29	0.03500
Laptm5	5:149775895-149797951	70.71	29.18	−1.28	0.02304
Mmp12	8:4249934-4328865	53.39	26.46	−1.01	0.04502
Ifitm2	1:201134356-201135537	141.63	72.01	−0.98	0.02873
Prl7b1	17:43783361-43791538	96.93	51.16	−0.92	0.04502
Sod3	14:63381447-63387180	7.22	20.37	1.50	0.00922
Tf	8:108196748-108244545	62.61	188.51	1.59	0.02304
Gpc3	X:139192114-139393977	9.56	30.69	1.68	0.00922
Ccdc37	4:124661801-124671607	5.76	18.58	1.69	0.00922
Cldn2	X:127538684-127549018	1.66	5.37	1.69	0.04862
Pcdh24	17:15937976-15962796	1.80	6.02	1.74	0.02873
Muc13	11:68772164-68794880	3.41	11.77	1.79	0.00922
Fgg	2:174727311-174734592	11.29	41.76	1.89	0.00922
Creb3l3	7:10106524-10114955	3.23	12.04	1.90	0.01702
Mttp	2:235613709-235654848	2.33	9.12	1.97	0.00922
Serpinf2	10:62748115-62756200	3.56	14.05	1.98	0.00922
Serpina1	6:127998618-128021719	4.90	20.49	2.06	0.01702
Fmo1	13:78503769-78536359	6.70	28.40	2.08	0.00922
Maob	X:17553528-17657839	6.15	26.17	2.09	0.00922
Rbp4	1:242443797-242450998	83.56	362.99	2.12	0.00922
Tdh	15:42758307-42771849	4.69	21.22	2.18	0.00922
Ttr	18:12406550-12413680	135.68	616.60	2.18	0.00922
Vil1	9:73748631-73776345	1.11	5.07	2.19	0.04502
Apoa4	8:49233139-49233436	61.26	291.64	2.25	0.00922
Apob	6:31508011-31556597	9.20	43.86	2.25	0.00922
Apoa2	13:87114733-87116372	70.35	335.96	2.26	0.00922
Spp2	9:87297051-87316545	16.85	80.97	2.26	0.00922
Apoc2	1:78979033-78980136	42.59	241.21	2.50	0.00922
Cubn	17:87655812-87772079	0.96	6.33	2.72	0.00922

*n* = 6 per group

**Fig. 1:**
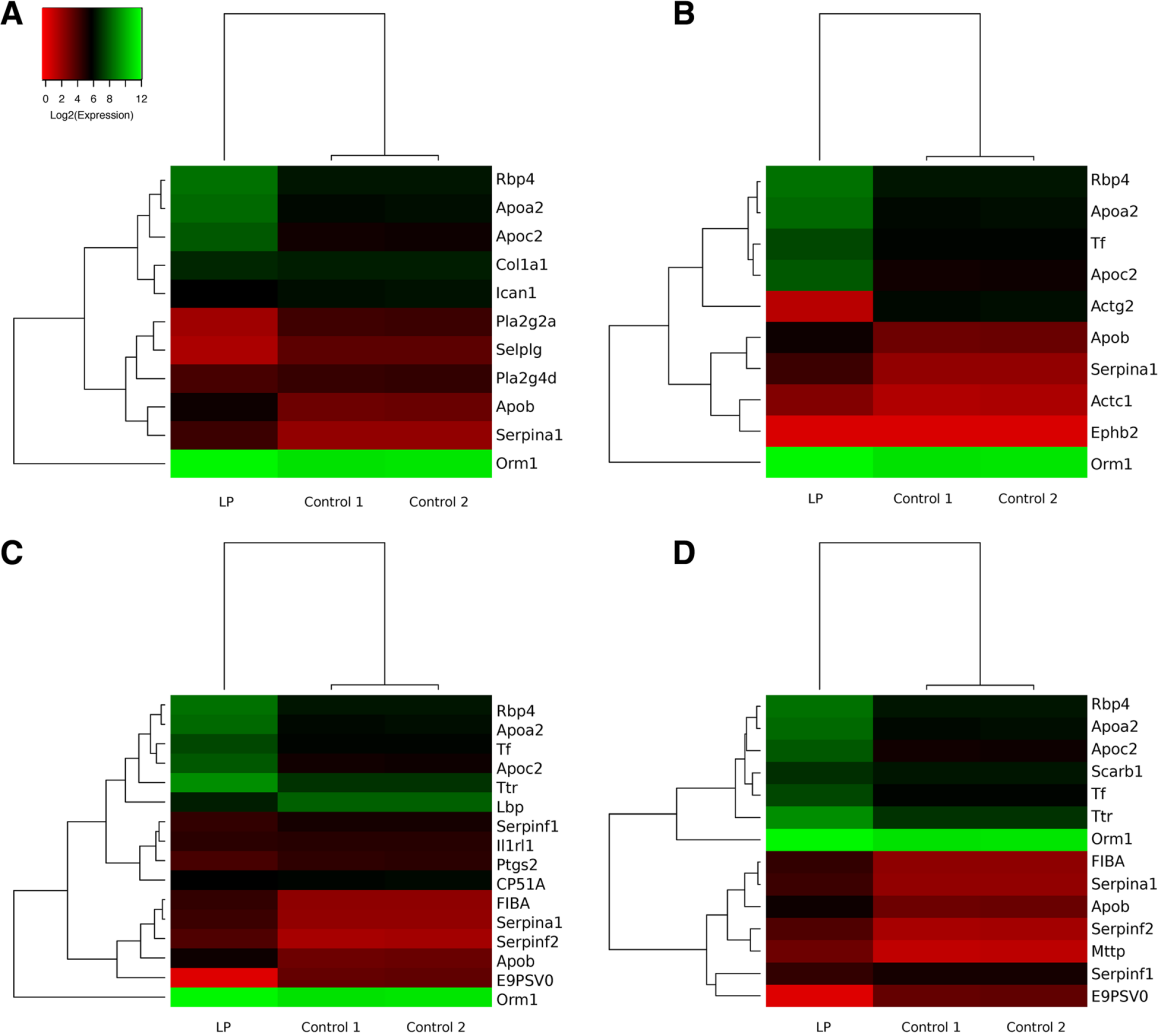
Heatmaps of gene expression (log2 FPKM) for pathways that are significantly influenced by maternal protein restriction. **a** Atherosclerosis signalling. **b** Clathrin-mediated endocytosis. **c** LXR/RXR activation. D FXR/RXR activation

Analysis of the data set using ingenuity pathway analysis identified 19 pathways that were significantly affected by maternal protein restriction with *P* < 0.01. A more stringent cut-off of *P* < 0.001 identified eight significantly affected pathways (Table 2). The top six pathways (acute-phase response signalling, FXR/RXR activation, liver X receptor (LXR)/retinoid X receptor (RXR) activation, complement system, atherosclerosis signalling, clathrin-mediated endocytosis signalling) were closely related functionally, with a strong focus on cholesterol uptake and efflux across the placenta. Figure 1 shows heat maps for the genes involved in the functionally interesting enriched pathways. A relatively small number of genes contributed to the enrichment noted for all of these pathways (Ttr, ApoA2, ApoB, ApoC2, Fgg, Rbp4, Serpin A1, Serpin F2 and Serpin G1).

**Table 2:** Pathways significantly influenced by maternal protein restriction in the day 13 rat placenta

Pathway	*P* value (log_10_)	Differentially expressed genes in pathway
Acute-phase signalling	11	C1R, C4A/C4B, Serpin G1, TTr, TF, C1S, ApoA2, Serpin A1, Serpin F2, Fgg, Rbp4
FXR/RXR activation	10.9	C4A/C4B, TTr, ApoB, TF, ApoA2, Serpin A1, ApoC2, Serpoin G2, Mttp, Rbp4
LXR/RXR activation	9.59	C4A/C4B, Ttr, ApoB, TF, ApoA2, ApoC2, Serpin A1, Serpin F, Rbp4
Atherosclerosis signalling	6.72	ApoB, ApoA2, ApocC2, Serpin A1, Pla2g2A, Selpg, Rbp4
Clathrin-mediated endocytosis	5.55	ApoB, RF, ApoA2, ApoC2, Serpin A1, Actg2, Rbp4
IL12 signalling in macrophages	4.08	ApoB, ApoA2, ApoC2, Serpin A1, Rbp4
Coagulation system	3.66	Serpin A1 Serpin F2, Fgg
Nitric oxide and ROS production in macrophages	3.47	ApoB, ApoA2, ApoC2, Serpin A1, Rbp4

The table shows ingenuity canonical pathways with significant enrichment in comparison of control and low protein exposed placentas

**Fig. 2:**
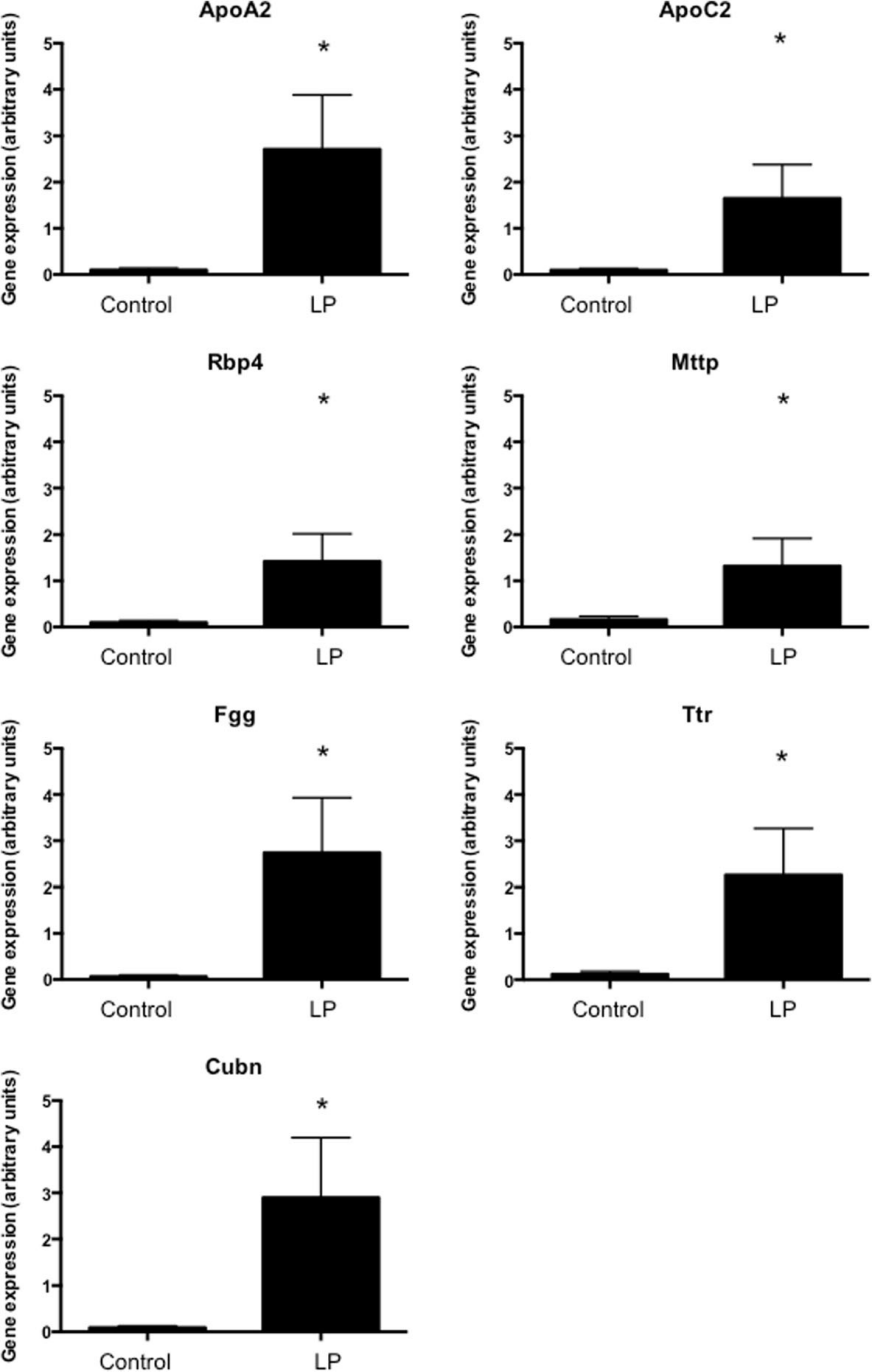
Expression of genes related to enriched pathways. Real-time qualitative PCR was used to validate the differential expression of seven genes related to canonical pathways identified by ingenuity as significantly influenced by maternal protein restriction. Expression was normalised to cyclophilin mRNA expression and *P < 0.05 between groups. *n* = 6 per group

**Fig. 3:**
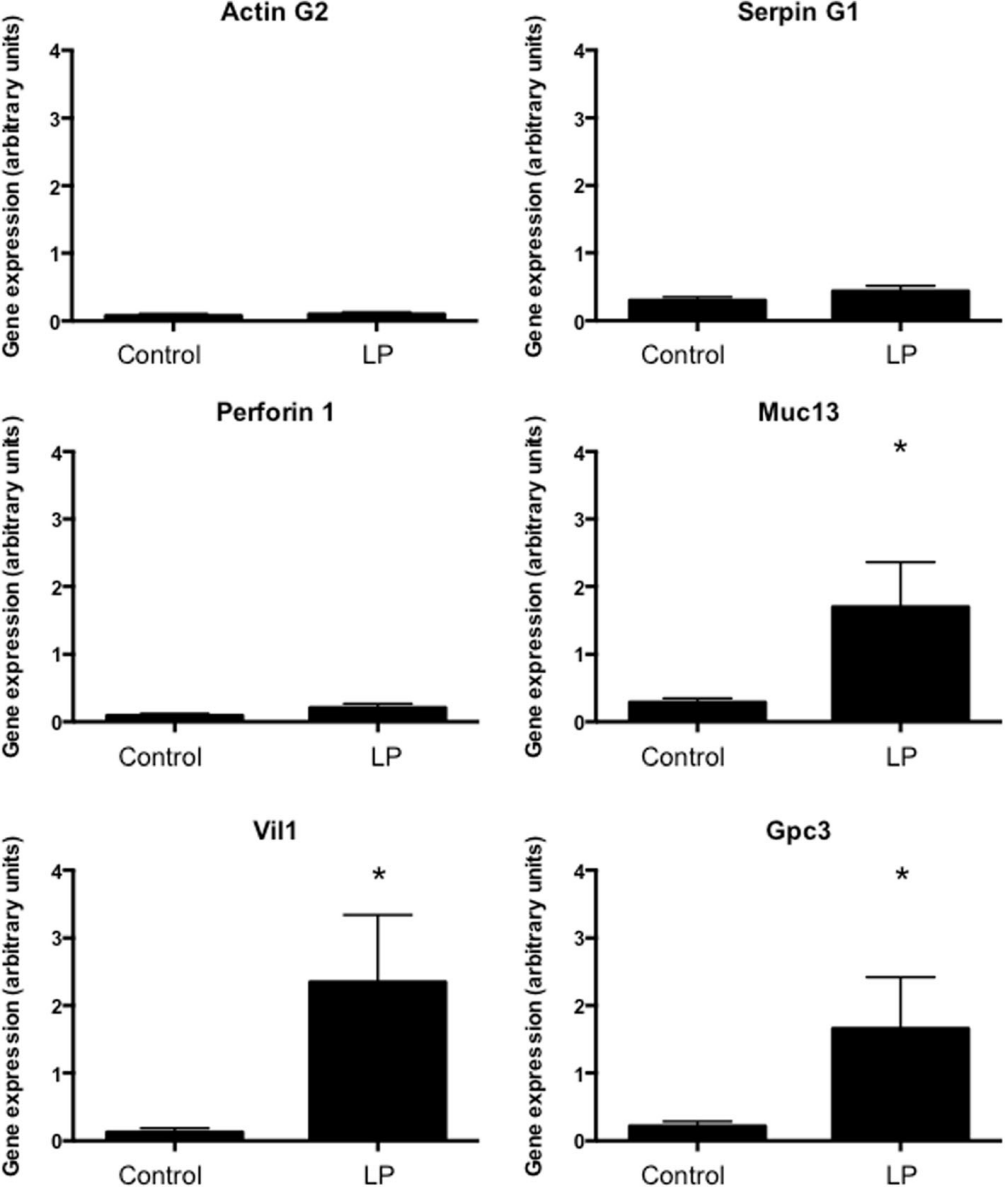
Expression of genes unrelated to enriched pathways. Real-time qualitative PCR was used to validate the differential expression of six genes related to canonical pathways identified by ingenuity as significantly influenced by maternal protein restriction. Expression was normalised to cyclophilin mRNA expression. *P < 0.05 between groups. *n* = 6 per group

**Fig. 4:**
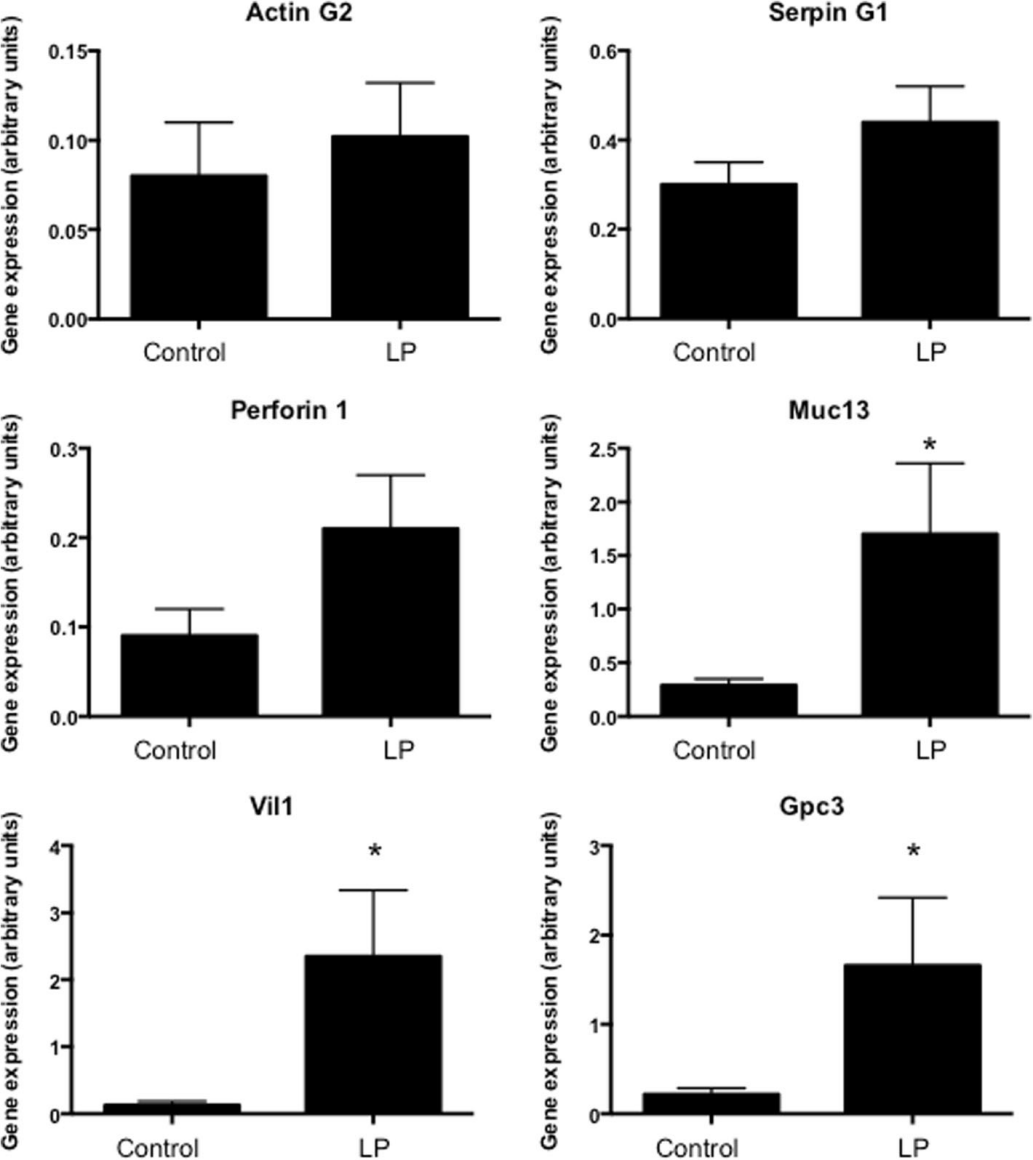
PCR analysis of three genes

To validate the observations made using RNASeq analysis, quantitative real-time PCR was performed to explore the expression of 13 genes in two selection groups. The first group comprised genes that were differentially expressed with protein restriction and deemed functionally significant (associated with cholesterol transport) based upon the Ingenuity analysis (Ttr, ApoA2, ApoC2, Rbp4, Fgg, Actg2). The second group were genes that were differentially expressed but not associated with the pathways identified by Ingenuity (Muc13, Vil1, Gpc3, Cubn, Mttp). It should be noted that Cubn has a role in the uptake of high-density lipoprotein (HDL)-cholesterol by the placenta and that Mttp has a role in the packaging of cholesterol and lipid into low-density lipoprotein (LDL). Figures 2 and 3 show the data from the PCR analyses of these genes and Table 3 compares the fold-change in expression noted in the RNASeq analysis. The majority of genes in the validation set were strongly over-expressed in placentas from protein restricted pregnancies compared to controls, with a minimum of 4.53-fold (Gpc3) and maximum 41.35-fold (Fgg) up-regulation noted in this set. PCR analysis of three genes did not reproduce the statistically significant effects of protein restriction that were shown by RNASeq (Actg2, SerpinG1 and Prf1; Fig. 4). The PCR analysis generally detected a greater degree of up-regulation in the validation set than was noted with RNASeq (Table 3).

**Table 3:** Comparison of fold-change in gene expression between RNASeq and real-time PCR

Gene	Log_2_ fold-change RNA Seq	Log_2_ fold-change RNA PCR
Actg2	−4.85***	0.28
Apo A2	2.26***	4.79*
Apo C2	2.52***	4.14*
Cubn	2.71***	5.01*
Fgg	1.88***	5.37*
Gpc3	1.68***	2.18*
Mttp	1.97***	3.14*
Muc13	1.79***	2.71*
Prf1	−4.04***	1.68
Rbp4	2.11***	3.85*
Serpin G1	−2.21***	0.69
Ttr	2.18***	3.52*
Vil1	2.19***	3.41*

Significant differences were noted between control and low protein exposed placentas within each analytical approach (*P < 0.05, ***P < 0.001)

## Discussion

In this experiment, we tested the hypothesis that maternal protein restriction would impact upon gene expression in the day-13 rat placenta. The data showed that this was in fact the case and that although the number of genes affected was small, the nutritional insult had a major impact upon expression of genes associated with cholesterol transport processes within the tissue. The expression of genes involved in the uptake of cholesterol by the placenta from HDL- LDL- and very low-density lipoprotein (VLDL)-cholesterol (ApoA2, ApoB, ApoC2, Cubn), the formation of clathrin-coated pits in which VLDL- and LDL-cholesterol receptors are located (Tf, Orm1, ApoA2, ApoC2, Actg2, Rbp4), the regulation of cholesterol efflux (Ttr, Tf, Orm1, Serpin F1, Rbp4, Mttp, Fgg, Serpin F2, Serpin A1) and the efflux from the placenta as LDL-cholesterol (ApoB, Mttp) were generally up-regulated by maternal undernutrition. Importantly, we have confirmed that the effects of maternal protein restriction during the first half of pregnancy may be mediated through changes in placental function.

Previous studies suggest that placental structure and organisation may be influenced by maternal protein restriction in both rats and mice [22–24]. These diet-related changes appear to be related to differential expression of adhesion molecules (beta catenin and vascular endothelial cadherin) and impaired cell proliferation. These processes appeared to be largely unaffected in the present study (although cadherin Cdh17 was down-regulated by protein restriction) and the discrepancies may stem from species differences or differences in stage of gestation at which samples were collected.

Functionally, placentas from protein-restricted rodents are known to differ in terms of materno-fetal steroid exchange [11] and transport of fatty acids and amino acids [12, 25, 26]. Whilst specific genes related to these functions have been previously identified as being sensitive to protein restriction, none were found to be differentially expressed in the current study. This is most likely explained by our study concentrating on day-13 rather than later stage placentas.

Cholesterol transport across the placenta is complex and involves a large number of proteins [27]. Cholesterol reaches the placenta in the form of LDL- VLDL- and HDL-cholesterol, which have ApoB, ApoC2 and ApoA2 respectively as their key structural proteins. LDL- and VLDL-cholesterol are taken up by their respective receptors which are located in clathrin-coated pits on trophoblasts. HDL-cholesterol can be taken up by SR-B1 (scavenger receptor class B member 1) or by binding to proteins such as megalin and cubilin. The latter two are multifunctional receptors which mediate uptake of material by endocytosis [28, 29]. Once taken up by trophoblasts, cholesterol is hydrolysed to cholesterol esters. Export from trophoblasts is in the form of either LDL-cholesterol or HDL-cholesterol. LDL-cholesterol is formed through placental expression of apoB and the action of microsomal triglyceride transfer protein (Mttp). HDL-cholesterol can be formed through complexing of lipids and cholesterol with a range of different apolipoproteins (ApoA1, ApoE, ApoA4, ApoC1, ApoC4; [27]). These are synthesised in response to LXR/RXR activation [30]. ApoA1 synthesis is also influenced by FXR/RXR activation [31]. Cholesterol efflux for formation of HDL-cholesterol complexes is dependent upon a range of ATP binding cassette proteins (AbcA1, AbcG1, AbcG5, AbcG8, [27]), which are downstream targets of FXR/RXR activation [32]. The present study has shown that almost all of these processes are sensitive to maternal protein restriction, and importantly, we have found that the only significant enrichment of pathways within our dataset lies in these processes. If there are any strong drivers of nutritional programming through the placenta at this stage of development, then cholesterol must play a key role.

The uptake of cholesterol by the embryo and fetus is critical for normal development [27], and defects of endogenous cholesterol synthesis are known to be lethal [33]. Cholesterol will also play an important role in placental function as it is the precursor for all steroid hormone synthesis. Disturbances of placental transport or endogenous fetal synthesis can have a number of effects on growth, cell proliferation, metabolism and the organisation of tissues [27, 34]. Low maternal cholesterol is associated with lower birth weight and microcephaly in humans [35], and women who have growth retarded infants have been found to have lower circulating cholesterol [36]. Optimal cholesterol transport to the fetus is therefore likely to have a positive impact upon development, and it is known that some of the effects are mediated through the cell cycle [37, 38]. However, some animal studies suggest that excessive cholesterol may also have a negative impact on growth. Bhasin et al. [39] reported that hypercholesterolaemia in pregnant LDL receptor knockout mice was associated with intrauterine growth retardation. The relationship between fetal cholesterol and the normal development and organisation of tissues may therefore be complex.

It is known that hypercholesterolaemia during pregnancy is associated with adverse health outcomes in the longer term. In humans, there is evidence that maternal hypercholesterolaemia is associated with the development of fatty streaks in fetal arteries [40], and cholestasis during pregnancy is associated with programming of an overweight, insulin-resistant phenotype in humans [41]. Animal studies have shown greater atherosclerosis in offspring of hypercholesterolaemic mothers [42, 43]. Previous work from our laboratory showed that in the ApoE*3 Leiden mouse, a transgenic rodent which has a predisposition to atherosclerosis, maternal protein restriction during fetal development increased atherosclerotic lesion size in adult life [44]. As atherosclerosis in this mouse is related to the degree of cholesterol exposure, it may be that intrauterine exposure to higher than normal cholesterol transport across the placenta may contribute to the adult disease phenotype. Induction of cholestasis using cholic acid in mouse pregnancy produces the same phenotype as in seen in humans [41] and is associated with greater cholesterol efflux from the placenta.

This study was an initial exploratory study to establish whether the placental transcriptome was significantly impacted by maternal protein restriction and to determine whether any observed effects were isolated to discrete processes within the tissue. One limitation of the study is that the whole placenta was used to generate the RNA, with no distinction between the maternal and fetal placental tissue. In the absence of any direct measurements of cholesterol transport or measurement of the genes of interest at the level of protein, assumptions are being made about the processes of cholesterol uptake and efflux being sensitive to maternal undernutrition. These measurements will be a priority for future studies, as will confirmation that placentas associated with female embryos respond in the same way as those from males.

## Conclusions

Current thinking about the mechanisms which link maternal nutritional status and long-term health in offspring is largely focused upon lasting epigenetic changes within the fetal genome [45]. This study has highlighted placental function as being modulated by maternal undernutrition and reinforces the alternative concept that programming of fetal development and long-term health may be a product of dysregulation of nutrient transfer across the placenta. Further studies are needed to evaluate cholesterol transport across the placenta in protein-restricted pregnancies and to determine the impact of cholesterol on fetal gene expression, epigenetic regulation of gene expression and tissue morphology. This analysis of the placental transcriptome at the point where the placenta is not fully mature has supported the hypothesis that maternal undernutrition impacts upon placental function. The findings of this study will provide a platform for further investigation of processes within placenta that may be important new mechanistic targets or biomarkers that indicate nutritional programming of disease.

## Additional files

Additional file 1: Table S1. Diets were prepared in our laboratory by mixing dry ingredients with the oil and then binding with water. The diets were then formed into balls which were dried at 60 °C for 24–48 h. Energy content of diets was determined by bomb calorimetry and protein content using a Flash Nitrogen analyser. (DOCX 22 kb)
Additional file 2: Table S2. Primer sequences for quantitative real-time PCR. (DOCX 23 kb)
Additional file 3: Table S3. Full transcriptome analysis. RNASeq data set, unedited. (XLS 6400 kb)

## Abbreviations

Actg2: Actin G2
Apo A4: Apolipoprotein A4
Apo B: Apolipoprotein B
ApoC2: Apolipoprotein C2
ApoE: Apolipoprotein E
CP: Control protein
Cubn: Cublin
Fgg: Fibrinogen gamma chain
FXR: Farnesoid X receptor
Gpc3: Glypican 3
HDL: High-density lipoprotein
Igf2: Insulin-like growth factor 2
LDL: Low-density lipoprotein
LP: Low protein
LXR: Liver X receptor
Mttp: Microsomal triglyceride transfer protein
Muc13: Mucin 13
PCR: Polymerase chain reaction
Prf1: Perforin 1
Rbp4: Retinol binding protein 4
RXR: Retinoid X receptor
Ttr: Transthyretin
Vil1: Villin 1
VLDL: Very low-density lipoprotein
Wnt2: Wingless-type MMTV integration site family, member 2
