# Supplementary material for: The effect of maternal undernutrition on the rat placental transcriptome: protein restriction up-regulates cholesterol transport
Source: Genes Nutr. 2016 Oct 12;11:27. doi: 10.1186/s12263-016-0541-3 (PMC5059985; doi:10.1186/s12263-016-0541-3)
Supplement: Additional file 1: Table S1. — Diets were prepared in our laboratory by mixing dry ingredients with the oil and then binding with water. The diets were then formed into balls which were dried at 60 °C for 24–48 h. Energy content of diets was determined by bomb calorimetry and protein content using a Flash Nitrogen analyser. (DOCX 22 kb) [file 12263_2016_541_MOESM1_ESM.docx]

**Supplementary Table 1**

|  | **Control** | **Low protein** |
| --- | --- | --- |
| Casein(g/kg diet) | 180 | 90 |
| Corn oil (g/kg diet) | 100 | 100 |
| Starch (g/kg diet) | 425 | 485 |
| Sucrose (g/kg diet) | 213 | 243 |
| Fibre (g/kg diet) | 50 | 50 |
| Methionine (g/kg diet) | 5 | 5 |
| Choline chloride (g/kg diet) | 10 | 10 |
| AIN 73 Vitamin mix (g/kg diet) | 5 | 5 |
| AIN 73 Mineral mix (g/kg diet) | 20 | 20 |
| **Energy (MJ/Kg)** | **20.22** | **19.68** |
| **Protein content (% diet)** | **16.59** | **7.95** |

Diets were prepared in our laboratory by mixing dry ingredients with the oil and then binding with water. The diets were then formed into balls which were dried at 60**°**C for 24-48 hours. Energy content of diets was determined by bomb calorimetry and protein content using a Flash Nitrogen analyser.
