# Supplementary material for: The effect of maternal undernutrition on the rat placental transcriptome: protein restriction up-regulates cholesterol transport
Source: Genes Nutr. 2016 Oct 12;11:27. doi: 10.1186/s12263-016-0541-3 (PMC5059985; doi:10.1186/s12263-016-0541-3)
Supplement: Additional file 2: Table S2. — Primer sequences for quantitative real-time PCR. (DOCX 23 kb) [file 12263_2016_541_MOESM2_ESM.docx]

**Supplementary Table 2: Primer sequences for quantitative real-time PCR.**

| Gene | Forward primer | Reverse primer |
| --- | --- | --- |
| Cyclophilin | TGATGGCGAGCCCTTGG | TCTGCTGTCTTTGGAACTTTGTC |
| Apoa2 | ACTGACTATGGCAAGGATTTGATG | CTCCTGTGCATTCTGAAAGTAAGC |
| Apoc2 | GAGCACTTGTTCAGTTACTGGAACTC | TGCTGTACATGTCCCTCAGTTTCT |
| Rbp4 | GAGGAAACGATGACCACTGGAT | TGCAGGCGGCAGGAATA |
| Mttp | TTTTCCTCTGTTTCTTCTCCTCGTA | AGCTTGTATAGCCGCTCATTATTTAAT |
| Fgg | CTGGCTGGTGGATGAACAAGT | TGGAGTAAGTGCCACCTTGGT |
| Ttr | CCGTTTGCCTCTGGGAAGA | CCCCTTCCGTGAACTTCTCA |
| Cubilin | TGCATGTCACCTTCACGTTT | TGTAAAGCCTCTCCCACTCC |
| Vil1 | AACCAGGCTTTGAACTTCATCAA | CGGACTCAGCCCCATCATT |
| Muc13 | CAGCAGTAGCACAGGTTCCAAT | CACAAGCAGAAGTAGCTGTCATACAG |
| Gpc3 | CGGTTTTCCAAGAGGCCTTT | GTAGAGAGACACATCTGTGAAAAATTCA |
| Serping1 | GACAGCCTGCCCTCTGACA | TTTCTTCCACTTGGCACTCAAG |
| Actin g2 | GATTGCTGACAGGATGCAGAAG | GGAGGAGCGATGATCTTGATCT |
| Prf1 | GCTGGCTCCCATTCCAAGAT | GCCAGGCGAAAACTGTACATG |
